# Brief admission by self-referral for adolescents who self-harm: discourses on involvement and responsibility among parents and other significant adults

**DOI:** 10.1186/s13034-025-00948-8

**Published:** 2025-09-23

**Authors:** Reid Lantto, Kajsa Landgren, Sophia Eberhard, Björn Axel Johansson, Olof Rask, Sofie Westling

**Affiliations:** 1 Psychiatry, Department of Clinical Sciences Malmö, Lund University, Lund, Sweden; 2 Department of Child and Adolescent Psychiatry, Skåne University Hospital, Helsingborg, Sweden; 3 Department of Psychiatry, Skåne University Hospital, Lund, Sweden; 4 Department of Clinical Sciences Lund, Division of Child & Adolescent Psychiatry, Lund University, Lund, Sweden; 5 Psychiatry, Habilitation & Aid, Child & Adolescent Psychiatry, Regional Inpatient Care, Emergency Unit, Region Skåne, Malmö, Sweden

**Keywords:** Brief admission, Self-admission, Self-harm, Parents, Qualitative, Discourses, Human rights, Responsibility, Involvement, Person-centered care

## Abstract

**Background:**

Brief Admission by self-referral (BA) was implemented in 2018 in Swedish child and adolescent psychiatric (CAP) inpatient care. This intervention empowers adolescents to self-admit at their own request for brief periods to prevent self-harm and suicidal crisis. As BA enhances healthcare user autonomy, it is a timely intervention to consider with the emerging human rights discourse and rising imperatives for person-centered care in psychiatry. The present study explores talk about adolescents access to BA specifically in terms of involvement and responsibilities of parents and other significant adults in CAP.

**Methods:**

In this qualitative study, we interviewed 26 significant adults (the majority being biological parents) of children with access to BA. Interviews were semi-structured, asking broadly about participants’ experiences with BA. We used reflexive thematic analysis from a social constructionist framework to explore how participants’ narratives drew on existing psychiatric discourses.

**Results:**

We constructed four themes around narratives of involvement and responsibilities in BA specifically and CAP generally: *there’s no need to be involved in BA*, *selflessly supporting child involvement*, *being insufficiently involved*, and *being left to shoulder everything*. These themes illustrate a sliding scale from perceiving little responsibility nor need for involvement, to perceiving shared responsibility for children’s well-being but limited personal rights, to being under-involved and even perceptions of being left with sole responsibility as CAP refused to provide care.

**Conclusions:**

Participants’ narratives could generally be mapped onto rights-based discourse, emphasizing that adolescents should have access to BA as it helped them care for themselves. As participants took various positions regarding the responsibility of parents versus CAP to protect and help adolescents, the risk of perpetually downplaying the needs of parents and other significant adults became apparent, along with the pitfalls of neoliberal healthcare management and responsibilization of child mental health. CAP ought to systematically inform both adolescents and significant adults about BA, strengthening mental health literacy among the target population. It also ought to be emphasized that supporting significant others is considered part of the purpose with BA.

## Introduction

Suicide and accidental death from self-harm behavior are among the leading causes of death in adolescents [1]. Self-harm behavior, with or without suicidal intention, is common among adolescents [2] and strongly predictive of future suicide attempts [3, 4]. While effective, evidence-based treatments of self-harm behavior exist, such as DBT-A, these are of limited and inequitable availability in child and adolescent psychiatry (CAP) in Sweden. In cases of acute suicidal ideation or behavior, children may be admitted to psychiatric inpatient care together with a parent or other significant adult. The aim is to keep these admissions short and, as far as possible, voluntary for the child.

Brief Admission by self-referral (BA) is a crisis management intervention currently offered in the southernmost region of Sweden to adolescents with recurrent self-harm and suicidal ideation. The overarching aim is to prevent self-harm while supporting the adolescent’s autonomy [5, 6]. The adolescent, a legal guardian, an outpatient therapist and the inpatient coordinator meet to discuss and agree on a plan (the BA contract), including conditions like maximum length (3 nights) and frequency (3 BAs/month) as well as individualized goals and coping strategies, early signs that the adolescent might need a BA, and specified needs during BA. When the adolescent feels they need a BA, they can call the inpatient unit and agree on a time of arrival. The BA contract is reevaluated every six months. As a rule, CAP inpatient care contacts a legal guardian whenever the adolescent utilizes BA. The BA contract is commonly incorporated into safety planning in inpatient and outpatient CAP, enhancing care integration.

As BA was originally an intervention for adults, research on BA for adolescents is still developing. A cohort study of 63 adolescents showed significant intraindividual decreases in emergency visits, psychiatric admissions, and days admitted after gaining access to BA, though episodes of coercive care remained unchanged [6]. In a qualitative interview study, adolescents with access to BA reported feeling safe, relieved, and empowered to self-admit without social stigma. Despite some challenges, BA was generally seen as helpful for managing crises and preventing self-harm [7]. However, implementation challenges have been noted concerning adaptation of an adult psychiatric model into a youth-focused system [8].

Surrounding these at-risk adolescents are parents and families caring for them, who often experience stress, guilt, shame, and personal health issues relating to the child’s self-harm, as well as not knowing how to help or talk to them [9–12]. Parents of adolescents who self-harm have reported a need for more information and better involvement in treatment planning and interventions [11, 13–18]. A phenomenological study on adolescent BA utilization identified two contrasting parental experiences: of safety, relief and trust as BA supported the adolescent’s recovery, vis-a-vis loss, confusion, and helplessness as parents struggled to adapt to new roles [19]. However, research is sparse on how parents and other significant adults make sense of CAP and their own responsibilities regarding child self-harm. A critical discursive research orientation enables engagement with the socio-political and historical contexts that shape these perspectives [20].

In recent years, global discourses about mental health have increasingly centered human rights. Rights-based discourse emphasizes personhood, autonomy and user participation. Individuals are construed as active rights-holders and agents of change in their recovery processes, having the right to healthcare and to consent freely to interventions. Accessible and anti-discriminatory healthcare services are promoted [21–24].

Rights-based discourse can be suggested to be manifested in Western healthcare largely with the movement towards person-centered care, again emphasizing participation and shared decision-making. Healthcare is considered person-centered when individualized, developed in partnership with the user and adapted to their personal resources and limitations. Person-centered care is considered a core competency for healthcare professionals, expected to respect and empower healthcare users [25]. These values are also reflected in Swedish law [26, 27]. In April 2025, the Swedish National Board of Health and Welfare emphasized its commitment to furthering person-centered care for individuals with severe psychiatric conditions by releasing online resources and training material targeting e.g. healthcare professionals [28].

BA, with its emphasis on autonomy, was developed as these discourses were on the rise globally and may be considered a human rights-oriented intervention. However, concerns are voiced that the discursive practice of promoting autonomy and independence under the guise of person-centered care may be used to legitimize the status quo of biomedical, neoliberal ideas governing psychiatry [29–32]. Biomedical discourse understands neurobiological processes and pharmaceuticals as central in mental health issues, rendering a deficit-based perspective of self-harm [33, 34] focused on e.g. difficulties with emotion regulation, distress tolerance and impulse control. Biomedical discourse is often related to neoliberal ideology, commercializing healthcare at the detriment of quality and health equity. Neoliberal discourse allocates responsibility onto the individuals to make appropriate choices, manage their own health and risks, and become productive members of society [29–32, 35–37]. There is some overlap, then, in the emphasis on individual autonomy between rights-based and neoliberal discourses, ‘rights’ being the key distinction. Critics caution that overly emphasizing individual autonomy may inadvertently allocate disproportionate responsibility onto the individual for their own healthcare, without appropriate regard for their circumstances and needs [29, 35, 36]. Arguably, adolescents may be more vulnerable to such risks.

In the southernmost region of Sweden, child and family participation is considered key for CAP inpatient care, but is only loosely operationalized as providing information, collaborating with stakeholders concerning treatment plans, and strengthening legal guardian caregiving [38]. Simultaneously, Swedish public discourse about mental health and psychiatry [39] identifies both children and parents as populations vulnerable to mental ill-health, while responsibility is allocated onto parents to foster resilience and prevent child mental illness. Psychological and psychiatric expertise is deemed necessary to support parents [39]. The challenging task of needing to navigate their own vulnerabilities while managing responsibilities puts parents in a unique position relating to neoliberal and rights-based discourses. In this landscape, BA actualizes timely questions regarding the needs, rights and responsibilities of parents and other significant adults in CAP.

### Purpose

The purpose of the present study is to explore how parents and other significant adults speak of their care involvement in relation to children’s access to BA. We seek to demonstrate how this can inform and complicate present understandings of care involvement and attribution of responsibility within CAP. Specifically, our research questions ask how parents and other significant adults negotiate their roles and responsibilities in the psychiatric care of children with access to BA, and what their narratives of involvement can tell us about implicit ideas of the responsibilities of present-day CAP in Sweden.

## Methods

### Design

We conducted this study from a social constructionist paradigm, where our role as researchers was not to uncover findings presumed to preexist in the natural world, but to generate meaningful knowledge on social reality. We were not concerned with ‘facts’ behind reported healthcare encounters, such as whether there was a plan for parental involvement in CAP, nor with participants’ evaluations of BA. Rather, we believed that participants’ accounts could tell us something about implicit ideas of CAP, how care involvement was constructed and where responsibility was attributed. We drew on Davies and Harré’s [40] writings on positioning and discursive practices to illustrate the dynamic and fluid positions that parents and other significant adults took in negotiating and understanding their roles and responsibilities.

We conducted in-depth, semi-structured interviews as part of a bigger project on the experiences of BA in CAP among significant adults, which rendered one previous publication on lived experiences of parents when their children self-admitted with BA [19]. The present study encompassed a larger sample, included voices of significant adults where children had or had not self-admitted, and explored discursive practices with involvement and responsibilities as its focal points.

### Setting

At the time of writing, the regional CAP inpatient unit in Malmö, Sweden, provided services to approximately 300,000 children and adolescents; approximately 90,000 were 13–17 years old. About 350 children and adolescents were admitted annually, of whom a large proportion presented with suicidal ideation. Since 2018, BA has been available at the inpatient unit as a crisis management method for adolescents with complex psychiatric care needs, recurrent self-harm and suicidal ideation, as previously described [6]. To be eligible for BA in its current form, the adolescent needed to have ongoing contact with outpatient CAP, at least one previous emergency admission or visit to the inpatient unit, and be expected to continue to utilize a high level of care. Since implementation, the number of adolescents with new BA contracts increased exponentially each year [41].

### Recruitment and data collection

First author RL identified significant adults via all BA contracts at the inpatient unit in December 2021 and phoned them to inform about the study and invite them to participate. They were also asked about other significant adults to invite. Those who were interested received written information via email and a follow-up phone call about two weeks later. We broadly requested significant adults’ experiences with BA, inviting a range of perspectives from low to high familiarity. We targeted and obtained perspectives of lay adults who had some personal connection to children’s access to BA.

The research team created and reviewed a semi-structured interview guide together with CA, a representative from the Swedish Partnership for Mental Health (NSPH), a non-governmental organization working to increase patient and family mental healthcare participation. RL conducted all interviews. A pilot was consecutively included in analysis. Interview questions were broadly formulated, encouraging contextualized descriptions rather than abstract theorization. The interviewer largely followed participants’ narratives. Neither in the recruitment material, nor during interviews, were topics of involvement, participation, responsibility, or person-centered care ever explicitly enquired. Interviews were to be held in private locations chosen by participants, though most were conducted via Zoom due to the covid-19 pandemic.

### Participants

Eligible for participation were all parents and other significant adults of children with access to BA at the regional inpatient unit. Other significant adults intended those who were taking active part in the everyday care of the child, such as stepparents, second-degree relatives, or even staff at service housing with insight into the child’s everyday life, who were able to relate to the child’s access to BA.

Out of 70 individuals invited to be part of the study, 26 significant adults of 23 children chose to participate. For nine participants, the child had never physically utilized BA, or the participant had no insight into admissions with BA. Seventeen were biological mothers, six biological fathers, one adoptive father, one foster father and one grandmother. Participants were between 37 and 75 years old, median age 48. For fifteen participants, there were other children in the household at least part-time. Participants were never asked about occupations, but volunteered information suggesting a broad range of job areas and socioeconomic situations, including healthcare, homecare, education, office jobs, seasonal jobs, construction, hourly jobs, and being retired. Some jobs involved lengthy commutes or frequent travel. The possibility of remote work varied considerably.

Six participants volunteered information about a personal history of mental illness, including self-harm, psychiatric admissions, and substance abuse. An additional few mentioned having significant others with such experiences, including siblings, partners and other children requiring extensive care. Nine participants reported current or previous contact with social services. Current or previous experiences with sick leave or lengthy leaves of absence to tend to the child were quite common.

### Analysis

We opted for reflexive thematic analysis [42–44], as the subjective approach to analyzing patterns and constructing meaning from data fit well with our social constructionist standpoint. Throughout analysis and reporting of results, we were guided by the quality criteria of Rigor, Resonance, Reflexivity, and Relevance [45]. RL was the driver of the analysis, with co-authors KL and SW involved in discussions of initial and revised codes and themes in meetings and written correspondence. These discussions addressed all quality criteria, though mainly centered on relevance and resonance.

We moved through the six phases of reflexive TA recursively. Data familiarization involved conducting, transcribing and rereading interviews, as well as making reflective notes and reflexive journals about the interviewer-analyst’s reactions to and interpretations of interview interactions. The bulk of analysis was done in Microsoft Word, including producing visual coding maps. RL used NVivo 14 complementarily, to gain an overview of codes and associated excerpts. Coding was more inclusive at first, then more specifically geared to the study aims. We were interested in both manifest and latent content.

RL generated initial themes by repeatedly reflecting on the overarching story in terms of discourses of involvement and responsibility attributed to various stakeholders. Naming and renaming themes necessitated further refinement, reconsidering whether themes were distinctive and meaningfully substantiated. In this process, we considered the metaphor of a theater performance and used it to compare various positions taken through the discursive practices of participants [40]. Traces of this analytic figment will be evident in the presentation of results. In reporting, we pseudonymized participant quotes to protect confidentiality while balancing readability and resonance.

## Results

We constructed four themes of qualitatively related yet distinct ways in which parents and other significant adults spoke of children’s access to BA, pertaining to their involvement and responsibilities in care: *there’s no need to be involved in BA*, *selflessly supporting child involvement*, *being insufficiently involved*, and *being left to shoulder everything*. A thematic summary of the discursive positioning of parents and other significant adults is presented in Table 1.

**Table 1:** Discursive positioning of parents and other significant adults: themes and examples

Theme	Metaphorical positioning	Example quotes
There’s no need to be involved in BA	Significant adults hold no responsibility as this is mainly allocated onto the child, with support from CAP. They voluntarily step out of the healthcare scene, cheering the child on from the spectator seats.	Agatha: *In reality*,* it’s the child who knows how they’re feeling. And the doctors are good at it*,* of course*,* but they really can’t go*,* like*,* inside of the child.* Claes: *While [the child] is [at the psychiatric unit]*,* I don’t think I need to keep tabs on her.*
Selflessly supporting child involvement	Significant adults share responsibility with CAP but downplay their own needs, only walking onto the stage in supporting roles while the child is the main character.	Petra: *Unburdening might be the wrong word*,* so I want you to understand it properly* […] *That’s not why you’re supposed to use BA*,* to* [slight laughter] *unburden [the parent].* Jonas: *As a parent […] the responsibility really is located with you […] to make sure that you have got the situation somewhat under control. But I still saw BA as an additional safety.*
Being insufficiently involved	Significant adults feel they take on a portion of CAP responsibility. They think they really ought to be involved as important characters themselves, but CAP is cutting their lines from the script.	Molly: *‘What the hell am I supposed to think as a parent*,* then*,* if I’m not allowed to be there? And you’re not taking responsibility for what she does while she is there?’ It feels insane.* Louise: *I’ve felt that I*, I *would’ve needed support*,* supportive talks because you’re carrying so terribly much and I* did not *have it in me.*
Being left to shoulder everything	Significant adults are compelled to take on full responsibility as they feel CAP steps out of the healthcare scene altogether. They are actors expected to perform prior to rehearsal, or theater critics pointing out that the whole stage setting is not working.	Holger: *I can feel like*,* that… That the [psychiatric] healthcare system is refusing*,* and they want to relocate responsibility onto loved ones to deal with these issues*,* instead of taking care of it themselves.* Lisbeth: *They wanted me to take her home.* […] *I told- ‘You*,* you’ve got to keep her*,* you’ve got to’* […] [voice breaks] *There are so many things I don’t really understand.*

### There’s no need to be involved in BA

One narrative among participants was that they were quite unaffected by the child having access to BA. Participants described a rather passive role of limited personal responsibility, fulfilled simply by supporting BA conceptually.

Agatha said they hadn’t spoken much about BA in the family. Despite actively engaging in the everyday care of the child, she hadn’t been involved in the child’s process of contemplating self-admissions. She clarified that whatever impression she had of BA was via the child:

Agatha: What [BA] means to me, it’s really that it feels […] very important to [the child]. […] Since it is so important [to her] to have that sense of safety that she feels, it simply is important to me, too. […] In reality, it’s the child who knows how they’re feeling. And the doctors are good at it, of course, but they really can’t go, like, inside of the child.

Agatha principally supported BA, though personally she was uninvolved, emphasizing the child’s own competency and responsibility and the collaboration between children and CAP.

Similarly, Claes, a father with very limited contact with his child, repeated throughout the interview that it was good that BA existed in terms of care availability and safety, primarily for the child. Personally, though, he appeared unaffected:

Claes: It’s been pretty much the same, I think, now as before. It’s just that I don’t have to […] drive [my child] there now because she has taken the train there on her own […] I don’t know if she’s been admitted lately, either. So I have no clue. And [her mother] doesn’t tell me much either.

Interviewer: Would you have wished to receive more information from the healthcare system, perhaps? Or how do you feel about that?

Claes: No, I really don’t feel like I have to, or like, that you need to know such things, as long as [the child] is feeling well, it doesn’t really [yawn] matter.

Claes repeated that he had not ‘been given’ information about BA, or other matters concerning the child’s wellbeing, yet firmly denied any expectation to be informed by the healthcare system, rather attributing that responsibility to his family. He further added that ‘while [the child] is [at the psychiatric unit], I don’t think I need to keep tabs on her.’ As such, BA was framed as mostly a matter between the child and CAP.

Ragnar, a foster father, was also happy with the child’s BA access and saw little need for personal involvement. On the contrary, he described that he or his wife had felt pressured to be there during BAs *as stand-in care professionals* to compensate for lack of CAP resources. Ragnar mused: ‘When it comes to biological parents, there’s like another- [sigh] other expectations of having rights and [mumbles] […] What can you expect from a foster home, is the question?’

In these narratives, significant adults are not suggested to have active roles or responsibilities in relation to BA. In the theater auditorium, they would cheer the child on from the spectator seats. Tying into this narrative, some participants said they didn’t have any personal needs regarding BA and/or that their needs were cared for elsewhere.

### Selflessly supporting child involvement

Some participants conveyed ambivalence when talking about their roles in BA and taking up space in the CAP setting. There was a strong notion that they needed to be selfless, like vessels for the child’s voice and autonomy. Their own degree of involvement with BA was not an end, but an instrumental means to support child involvement and wellbeing. As such, they appeared to struggle to center themselves in the interview and put their own needs into words, frequently responding to questions about *their* perspectives by describing (their perceptions of) the child’s experiences: ‘She thinks it’s so-so, herself’ (Emelie), ‘*Her* thoughts go in a loop’ (Bo, emphasis added), ‘*She* likes having the opportunity’ (Sofie, emphasis added), ‘It must feel really nice to know that “I can get in whenever”’ (Beatrice).

There were accounts of actively working to conceal and push aside their own emotions to hold space for the child, not reacting when facing setbacks, mechanically patching the wounded child up ‘like a machine’ (Lina), feeling ‘almost heartless’ (Jonas) when doing so. As Josephine put it, ‘when the kids are with me, I am 100% mother all the time, and then there isn’t really space for what I think or feel or want.’ Sometimes, participants related this to feelings of guilt, claiming responsibility for the child’s current state and care.

In this context, addressing their own needs meant justifying them by reference to the child’s best interests. Personal benefits of BA tended to be framed as fortunate coincidences, as the child was the *real* target of the intervention:

Petra: Unburdening might be the wrong word, so I want you to understand it properly […] That’s not why you’re supposed to use BA, to [slight laughter] unburden [the parent] […] [but] when I’m not getting unburdened, I’m not good for [my child]. So it does fulfil like a positive function beyond me resting up, which is that when [my child] comes back home, I […] can sort of be better for her.

Notably, Jonas emphasized that he recognized that parents were largely responsible for their children’s wellbeing, and that BA was meant for the children:

Jonas: As a parent […] the responsibility really is located with you […] to make sure that you have got the situation somewhat under control. But I still saw BA as an additional safety in, like, this difficult hardship. […] As a parent to her, I feel that [BA] is a ***lovely***, lovely thing to be able to offer those kids.

In these narratives, participants cautiously suggested they may be affected by children’s access to BA and may have their own needs in relation to this, yet it was also clear that they wouldn’t expect to be considered in their own right. Significant adults could only walk onto the theater stage in supporting roles.

### Being insufficiently involved

This narrative contrasted with the previous theme as participants *did* expect to be involved in BA, as with any other process in CAP. They also expected to have their own needs considered. Their descriptions conveyed disappointment with an inappropriately passive CAP. Rather than receiving an invitation into collaborative care, participants described needing to fight for involvement, for their child and themselves.

For instance, participants reported that they were not properly informed about BA. Nellie described that even as the child was offered BA, parents were the last to know: ‘We weren’t really involved in that process. […] [The psychologist] just asked if we wanted that opportunity, and [our child] had said yes.’ This lack of involvement left participants searching for information from friends, acquaintances, the internet, and parents with similar experiences. Mikaela couldn’t believe that information about BA, and other relevant mental healthcare information, wasn’t systematically communicated to families:

Mikaela: You could imagine this general, like, information meeting [brief laughter] that would go like, ’Yes well, psychiatry is constructed this way and there’s outpatient care and mid-level care where you can receive this’ […] I have a friend who’s a nurse […] she said like ’but next time you go for a meeting [at CAP], ask for these and these things.’ […] If she hadn’t said that it never would’ve come up. [laughter] And that feels completely mental, like, when you think about it structurally!

Participants had doubts about certain conditions for BA, most commonly that the child would be free to leave the unit. They felt the parental perspective wasn’t appropriately considered. Molly reported openly objecting to this condition:

Molly: We’re talking a kid who self-harms and has [laughter/scoff] ***very*** pronounced clear suicidal thoughts that pop up ***all*** the time. Should I let her go to [the big city where BA is offered], where she is free to roam the streets by herself at any time? Which I would have never let her do, myself. And when I bring this up and say, ‘so what do we do if she wants to go out?’ Yes, no, they couldn’t stop her then, but then I’m saying ‘what the hell am I supposed to think as a parent, then, if I’m not allowed to be there? And you’re not taking responsibility for what she does while she is there?’ It feels insane.

Despite elaborate protests, Molly still had no power to renegotiate the terms of BA.

Petra explicitly suggested that the ideal of promoting children’s rights to autonomy and integrity clashed with and trumped parental inclusion in CAP, rendering parents under-involved or excluded:

Petra: Sometimes the healthcare system forgets that, parents have an important role […] We’re talking about minors so we, like- They should really get to feel heard, but us parents, we must also be heard and be, like, involved in,* in*, the care plan more closely. […] [Our child] gets an incredible amount of time in private where they, where the healthcare system sort of sends us [parents] out of the room. […] We would need to hear part of what she says in there to be able to do our job as parents. […] There’s something there about, also, the view of parents, with respect to the child’s rights.

Finally, participants conveyed unfulfilled expectations of personal support from CAP. As Lina put it, ‘You’re not offered a whole lot when you have a child who is mentally ill, in terms of support.’ Louise explained why it was crucial for CAP to actively offer parental support:

Louise: I’ve felt that I, ***I*** would’ve needed support, supportive talks because you’re carrying so terribly much and I ***did not*** have it in me. And I’ve gotten in touch with this therapist now, because in the middle of it, I did not have it in me. You’re so ***extremely*** focused on the children! So even going to the dentist for your own sake, everything gets deprioritized! It’s really just the children. So making that contact myself right in the middle, it just wasn’t on my map.

When asked if she would have been able to receive support at that time, Louise responded affirmatively as it felt necessary ‘to air it out’, but she would’ve rather had continual counselling scheduled. Bo noted how being offered support in the context of BA could be important ‘in case you’re not receiving it elsewhere within the healthcare system.’

In sum, in this narrative, active involvement and consideration of parents and other significant adults was construed as an obvious goal, or duty, for CAP. Participants were rather disappointed with insufficient efforts to secure this, much like when important theater characters were having their lines cut from the script.

### Being left to shoulder everything

Finally, in another narrative, some participants related to the child’s access to BA as missing the target, frequently widening the perspective to the healthcare system at large. Participants spoke of a system that not only failed to involve them, but checked out on them altogether, leaving parents solely responsible for the survival of their children. Some stated that the general healthcare system lacked resources and faced cutbacks, understanding BA as a budget option compromising care quality. Parents stated that their children needed treatment, which was out of the parents’ hands, yet they needed to step into such roles in the absence of a functional CAP.

Holger: Well, I’ve been in with [my child] in the [somatic] emergency room at least five times. And… Sure, they take care of the acute conditions. I can feel like, that… That the [psychiatric] healthcare system is refusing, and they want to relocate responsibility onto loved ones to deal with these issues, instead of taking care of it themselves.

The sense of refusal of CAP to provide care appeared to baffle Holger. He went on to talk about the importance of knowledgeable people on the case, implying the need for specialized knowledge to provide appropriate care:

Holger: It’s completely incomprehensible! They write on their [public healthcare] webpages, for us to do this and that, but then when you do that, you’re met with nothing. […] The healthcare system and psychiatrists and doctors, they are the ones who are supposed to be the experts. I’m not supposed to, be that. […] I have always, like, pushed back and told them, ‘But this can’t possibly be right. We can’t take her home now. She is trying to kill herself. All the time!’ ‘Oh but surely you can handle it.’

Other participants made similar statements that parents and significant adults lack the appropriate knowledge, resources or ability to care for children who self-harm at risk of suicide. Lisbeth relatedly described being left with sole responsibility in a situation that felt unmanageable:

Lisbeth: The police has driven her [to the psychiatric unit]. […] [My child] is high as a kite. [2 s pause] And they didn’t want to keep her, they wanted me to take her home. And I tried to explain that I have- There are younger siblings at home, […] she’s hysterical […] she had taken pot, benzo, amphetamine, she really was [emphasizing every word] high. As. A. Kite. And she reeked of alcohol. And they wanted me to take her home. […] I told- ‘You, you’ve got to keep her, you’ve got to’ […] [voice breaks] There are so many things I don’t really understand.

Participants reported being left to deal with matters such as assessment, treatment planning, crisis support, and generally keeping the child alive. Sometimes they addressed these situations as unreasonable and resisted in various ways.

At times, participants’ criticism was explicitly directed at BA, describing that BA wasn’t delivered as promised, prompting participants to take matters into their own hands. Mostly, however, participants criticized CAP more generally and seemed to make sense of BA by relating it to other forms of care within CAP, conveying a sense that obtaining BA did little to counteract broader issues. This narrative could be represented by actors being expected to perform prior to rehearsal, and/or the theater critic exclaiming ‘never mind the lines; the stage is wrong!’

## Discussion

The present article sought to explore how significant adults spoke of their care involvement in children’s access to BA and where responsibility was attributed. We constructed participants’ narratives on a sliding scale from perceiving little responsibility and no need for involvement, to perceiving a shared responsibility with CAP but limited right to personal support, through to narratives of being under-involved and, finally, being inappropriately left with sole responsibility facing perceived CAP pushback.

The first theme, suggesting contentment with low involvement, could be said to map onto rights-based and person-centered discourses of mental healthcare [21, 24, 25], emphasizing that BA was an important psychiatric intervention that these children couldn’t do without, while conveying faith in children’s self-insight and competency to recognize their own needs. These results appear to partially contradict the Swedish public discourse that parents are responsible for their children’s mental well-being, though in need of support from mental health professionals [39]; rather, participants conceptualize BA as a form of mental health support which enhances children’s autonomy and capability of caring for themselves, shifting the bulk of responsibility from parent to child. Psychiatry is still there to offer a care setting, though much responsibility rests on the child to seek care on time and use own coping strategies while admitted. This transfer of responsibility from parent to child is aligned with neoliberal healthcare ideals of early autonomy and individual accountability, nearly making the child solely responsible for their health [32, 36], though it could also be understood as a successful implementation of person-centered care, enhancing the child’s freedom and competency [25].

The second theme clearly conveys children’s right to healthcare and respect for their experiences and autonomy, echoing rights-based discourse [24], though the parents aren’t understood to be rights-holders. Their selfless position, where needing to occasionally center oneself seems forbidden, could be considered a role conflict, understanding helper versus help-seeker roles to be mutually exclusive. CAP research frequently stresses parental involvement while discursively conceptualizing parents as *resources* to support child well-being [46–48]. Immediate orientation to the parent-as-helper could risk precluding consideration of parents’ own needs, reinforcing a dichotomous perspective where parental needs for support and unburdening would be inherently suspicious and shameful. Previous research suggests that shame and guilt are among the reasons why parents hesitate to seek support for themselves [10, 11] despite extensive documentation of the need for clinical support [10, 12–14, 16, 17]. Crucially, without built-in structures to systematically offer information and support to significant adults who may have trouble voicing their needs, CAP risks missing the conversation, inadvertently contributing to the notion of parents as parenthetical. In other words, CAP risks falling into a perpetual cycle of downplaying these stakeholders, at worst stigmatizing ‘needy’ parents as inadequate helpers.

Importantly, descriptions of needing to discover crucial information about BA on their own can be conceptualized as shortcomings of CAP in securing the child’s right to health [21], placing disproportionately high demands on mental health literacy for parents and other significant adults [49]. This criticism highlights some flaws of neoliberal healthcare discourse and management. Interestingly, health literacy overall is increasingly being framed as a social issue, a matter of health equity, for which healthcare systems are recognized to share responsibility [50]. Notably, one participant’s comment about the structural perspective revealed an idea of the current modus operandi as one where CAP was not taking sufficient responsibility for providing equitable healthcare opportunities. In such objections, participants appear to draw on a rights-based discourse [21, 24].

Another key point was the perceived tension in CAP between facilitating parental involvement and protecting child integrity and autonomy. Interestingly, in this context parents are constructed as rights-holders along with the children and were thus able to resist and challenge under-involvement. This was not to say that participants questioned the dominant discourse that parents are chiefly responsible for their children’s mental well-being [39]; rather, parents could take on that discourse to insist that they would not be effectively mobilized to fulfil their responsibilities as long as they would remain largely excluded from the child’s healthcare processes. This response accentuates one problem with current operationalizations of person-centered care: unless children and parents are considered co-stakeholders in the child’s care, CAP risks allocating disproportionate autonomy and responsibility onto the child and overlooking their care needs– a hallmark of neoliberal ideology, which privileges autonomy at the expense of structural support [32, 36]. This risk might be amplified in the case of BA, an intervention which more explicitly aims to enhance the autonomy of the child. Participants in the present study appeared to resolve this issue by constructing parents as simultaneous rights-holders and responsible parties in the child’s care while preserving children’s interests and participation– a balancing act that is increasingly recognized in research [48, 51] and clinical perspectives on BA [47].

Though recurrent self-harm behavior and elevated suicide risk are formally considered to warrant CAP attention, in the fourth theme participants perceived pushback where CAP appeared to regard these matters as universal human experiences remaining up to the individuals (or in this case, their parents) to handle [39]. This could be considered a form of responsibilization, a process associated with neoliberal politics in which certain responsibilities previously carried by the state or other high authority are shifted to the individual or community (52; see also [35] about risk management). To put this in context, Sweden has a public healthcare system which is provincially governed and subsidized for healthcare users, with a strong legal and public notion that healthcare should be equitably available to everyone (see Chap. 3, 1§ of the law of health and medical care, [26]). Participants in the present study construed mental health professionals as bearers of a neoliberal discourse eroding care. Parents explicitly challenged this discourse, rather relentlessly arguing that these premises cannot be right, reframing the issue as psychiatric and insisting that CAP takes (back) responsibility, implicitly appealing to children’s rights.

Of note, while the present article has focused on the macro perspective of global rights-based and neoliberal discourses on psychiatry, participants appear to be drawing on Swedish cultural values and family norms in their understandings of BA and responsibilities in CAP. Sweden is an individualistic country where autonomy and independence are strongly valued, along with privacy and emotional restraint, and where trust in institutions is high as compared to the rest of the world [53, 54]. Institutional trust, respect for privacy and fostering independence may provide useful interpretive lenses for understanding the withdrawal of significant adults in the theme about no involvement, coupled with the value of emotional restraint in shaping the selflessly supportive parental vessel. This aligns with research suggesting that in more individualistic cultures, parental self-efficacy generally deteriorates during the child’s adolescence [53]. A sense of violated trust may also inform interpretation of strong reactions in the themes about insufficient involvement and shouldering everything. Culture appears to have shaped the locally situated expressions of global discourses.

### Clinical implications

The present study brings to light that having a child with access to BA seems to raise adequate questions, concerns, needs for support as well as wishes for involvement among parents and other significant adults. The regional CAP care is generally family-focused, requiring parents to be present during admissions and involving them in decision-making and supporting their child. In contrast, the more exclusive focus on enhancing the child’s autonomy in the current framework of BA seems to leave parents and significant others behind. The accounts of those who described a need to fight for being involved make apparent that there is no natural recipient for them to turn to, nor a clear procedure to secure that they are invited to be part of the adolescent’s BA process.

BA was originally developed for adult psychiatric patients, intended to strengthen their autonomy to be their own advocates while navigating the frontier between in- and outpatient care. When adopting the method for adolescents, emphasis was put on keeping aspects of autonomy also for them, without jeopardizing safety. It appears that this potentially contributed to downplaying the importance of structured involvement of significant adults in the intervention. Crucially, strengthening child autonomy with respect to CAP should not inherently imply strengthened autonomy with respect to significant adults.

An obvious clinical implication would be to secure structures for involvement and support for significant others as part of the intervention, for example by openly inviting (several) significant adults to dialogues about BA, as well as securing that they are offered individual support and guidance, if applicable and appropriate. In offering, negotiating, providing and following up on BA, remembering to ask about the needs and experiences of significant adults might go a long way. Actively de-stigmatizing parental needs would be recommended, for instance by communicating that providing relief and support to significant others has always been part of the purpose with BA [55], which is mirrored in recent Swedish research [7, 56]. Additionally, securing systematic, equitable access to information material on BA ought to be prioritized to strengthen mental health literacy among the target population, including families. When it comes to verbal information, CAP professionals might strive to inform adolescents and parents at the same time; when this isn’t possible, professionals may seek to provide a rationale explaining why, in keeping with respecting individuals’ preferences which is a key feature of the BA method. In much of these efforts, continued dialogue between in- and outpatient care may prove beneficial.

What’s clear is that one size doesn’t fit all when it comes to involvement, and professionals must work to balance the interests of all parties flexibly. Contextual considerations must be made continuously, such as whether, how and to what end parents and other significant adults feel the need to be involved, as well as their resources, capacities, and potential hurdles complicating involvement. For instance, parents who encourage BA or occasionally support their adolescents in making requests don’t necessarily *limit* the adolescent’s autonomy; rather, this could be key to them developing autonomy, much like parental scaffolding in other areas. However, parents who would make it a habit to determine the child’s needs and make decisions for them might need professional reminders of the aim of BA, preferably in collaborative conversations about helpful parenting roles over the course of the child’s BA process and general development. Similarly, if parents feel excluded– or concerned about safety– in cases when adolescents prefer them not to be present during BA, rather than perpetuating a dichotomous perspective where child and parental autonomy and involvement are in opposition, professionals might work collaboratively to include an agreement in the BA contract about how the adolescent, family and CAP will communicate during BAs.

Another narrative addressed perceived major shortcomings regarding both CAP and the healthcare system at large. Having access to BA, a crisis intervention, will never be able to compensate for major shortcomings in CAP. The discouraging testimony from struggling parents deserving much better is a sharp reminder that quality of CAP needs to be closely monitored and prioritized by decision-makers in healthcare. Finally, conflicting ideas about the purpose and governance of CAP and psychiatry at large, as evident in constructions of (and resistance against) neoliberal psychiatric praxis, ought to be addressed for Swedish CAP care to truly move toward person-centered and rights-based care [21, 24, 28, 36].

## Strengths and limitations

Throughout this research process, we have been guided by the quality criteria of Rigor, Resonance, Reflexivity and Relevance [45]. Rigor is about conducting the research process in a competent and systematic manner, and we believe we have collected data and worked through the analysis in such depth as to generate meaningful, coherent and distinct themes, each revolving around their own core organizing concept [42–44] and supported by participant quotes.

Resonance is about producing an evocative, powerful, compelling story [45], to which end we have strived to select telling and resonant interview extracts and frame them in such a way as to bring out nuances and complexities, with respect for participants’ perspectives and circumstances.

Reflexivity is about considering (and working with) our own subjectivities throughout the research process [45] and engaging actively with the research project from a position of knowingness [42]. We have attempted to be transparent about our choices and rationales throughout reporting. In this context it should be mentioned that this research team comprises a diversity of academic and clinical perspectives on self-harm (including medicine, psychology and nursing) as well as comprehensive clinical experience in the fields of self-harm and suicidality. Additionally, all members of the research team approach this research from some level of personal commitment to the topic, including lived experience with self-harm and experiences as significant others. These positionalities, while not disclosed to participants, have certainly influenced interview conversations as well as our understanding and interpretation of what participants said. Other authors conducting similar studies would likely have taken them in different directions.

Finally, relevance is about the contribution and applicability of the study [45], which we believe we have demonstrated specifically in discussing the important clinical implications for further development of the BA method and delivery in CAP, as well as broadly in terms of complicating the perspectives on involvement, responsibilities and what rights-based, person-centered care entails in CAP.

Beyond the four R’s [45], a notable strength of the present study is the richness of our interview material. We invited all members of this population that we were able to reach. By not explicitly enquiring about perspectives on involvement, responsibility, participation or person-centered care during recruitment, we believe we were able to recruit a more diverse sample who were personally affected by BA to various degrees and obtain their contextualized accounts on these matters. The diversity of the sample largely appears to reflect the population of (known) significant adults to adolescents with access to BA in this region, supporting transferability.

The clearest exception to this statement, and a major limitation of this study, is that we were unable to contact three listed significant adults who did not speak Swedish nor English, meaning that the perspectives of racially and ethnically minoritized populations in this area remain underexplored. Arguably, cultural factors as well as class, race/ethnicity and migration status may influence perceptions of responsibility and involvement in care. We know that there are structural barriers to care for marginalized populations, as evident in e.g. the underrepresentation of non-white Swedish adolescents holding a BA contract. Interviewing individuals from racially and ethnically minoritized populations who likely already face difficulties accessing services, would probably generate different accounts from those presently reported. For the present study, we considered employing translation services as well as hiring interpreters for the two languages in question. Regrettably, after months of discussion and preparation, we eventually decided it was not feasible due to logistical barriers and lack of resources.

Another limitation is that it wasn’t always straightforward to determine whether participants spoke about BA; sometimes they seemed to confuse BA with other interventions in CAP or even social services and would ask the interviewer for clarifications, stating that the child had been through so many processes that keeping them apart was difficult.

Further, at times it proved challenging to maintain our aims and even our epistemological underpinnings– especially when sitting with evocative, phenomenologically oriented descriptions of powerful personal experiences– though we believe we managed to appropriately return to broader constructions of social meaning.

## Suggestions for future research

It would be relevant for future research to adopt an intersectional lens in exploring experiences of BA among non-Swedish, non-English speaking populations specifically, and possibly among other multiply marginalized communities, who tend to be underrepresented in research as well as underserved in mental health settings. Recommendations for inclusive research design ought to be considered, such as working with interpreters, possibly matching interpreter-participant characteristics if relevant for the issue at hand, offering training sessions to familiarize interpreters with the topics explored, ensuring that researchers are familiar with navigating tripartite communication [57], and especially considering the relational dynamics of power and trust implicated in such conversation settings [58]. Interview questions should explicitly engage with cultural issues, e.g. being informed by the Cultural Formulation Interview of the DSM-5 [59] and should be attentive to experiences of discrimination and social stigma [60].

## Conclusions

Participants’ narratives largely align with the human rights discourse in mental health, while highlighting some pitfalls of neoliberal healthcare management. The roles of significant adults concerning BA in CAP may fluctuate over time and context. Efforts to strengthen their involvement will need to be sensitive to such fluctuations, with healthcare professionals paying continual attention to the personal circumstances of all stakeholders.

Participants recognized CAP as pivotal in providing support and resisted responsibilization processes seeking to displace disproportionate responsibility onto children or parents. As narratives suggested that CAP doesn’t sufficiently address how enhancing the adolescent’s autonomy can be balanced with protecting their safety, enhanced, solution-oriented collaborative discussions may be advised, involving all stakeholders. These may be initiated during BA contract negotiations, at intake or discharge conversations, or possibly in communication with outpatient care, with emphasis on ongoing dialogue rather than singular checkbox approaches. Flexibility is key in responding to the needs of all stakeholders.

The BA intervention crystallizes the complexities of care involvement in CAP and provides a suitable context to further develop professional work in these areas, the insights from which may benefit CAP at large.

## Data Availability

No datasets were generated or analysed during the current study.
